# The role of BTG1 and BTG2 genes and their effects on insulin in poultry

**DOI:** 10.3389/fphys.2024.1315346

**Published:** 2024-01-31

**Authors:** Egor Igorevich Kulikov, Lidia Ivanovna Malakheeva, Alexey Sergeevich Komarchev

**Affiliations:** Federal Scientific Center, All-Russian Research and Technological Poultry Institute, RAS, Sergiyev Posad, Russia

**Keywords:** BTG1 gene, BTG2 gene, insulin, chicken, metabolism

## Introduction

In recent years, there have been many studies that have shown the direct and indirect effects of B-cell translocation gene 1 (BTG1) and BTG2 expression levels on insulin secretion and sensitivity of the cell to insulin. These genes belonging to the B-cell translocation gene/transducer of the BTG/TOB family are expressed in different cells and regulate cell cycle processes, such as proliferation, apoptosis, and differentiation (Fletcher et al., 1991; Mauxion et al., 2009; Weisel et al., 2010). They were highly specific among the species of living organisms (Rouault et al., 1993). These studies suggest that there is a direct correlation between the expression levels of the BTG1 and BTG2 genes, standard of insulin, and metabolism in broiler chickens. The study of these genes and their influence on metabolism is, therefore, a promising area of research for physiologists and geneticists.

## The role of insulin in poultry

All metabolic processes occurring in a living organism are based on the use of energy in the form of ATP, synthesized mainly from glucose. As with all metabolic processes, metabolism of glucose is compiled by neuro-humoral regulation and participation of hormones such as insulin and glucagon. Insulin is synthesized in the β-cells of the pancreas (Moore and Cooper, 1991; Norton et al., 2022). Secretion of insulin is related with the level of metabolism in broilers (Vertiprakhov et al., 2023). Tissue metabolomics indicated the unique effects of insulin on amino acid metabolism (Cai et al., 2012).

Insulin is a hormone produced by the pancreas in response to high blood glucose levels. It plays a crucial role in regulating glucose metabolism in the body. In chickens, insulin has been shown to have several effects, including the following:

1) Glucose uptake: insulin stimulates the transfer of glucose from the bloodstream into cells. In chickens, this is particularly important for muscle cells, which use a lot of glucose for producing energy during movement and growth (Qaid et al., 2016).
2) Lipid metabolism: insulin also regulates lipid metabolism in chickens by promoting the storage of excess fat in the adipose tissue and inhibiting the breakdown of stored fat for energy (Saltiel et al., 2001).
3) Growth and development: insulin is a key regulator of growth and development in chickens, particularly during the early stages of life. It promotes cell division and differentiation, leading to increased muscle and bone growth (Hill et al., 1985).
4) Reproduction: insulin also plays a role in reproductive function in birds by regulating the production of steroid hormones and supporting the development of oocytes (Sliwowska et al., 2014).

Overall, insulin is essential for maintaining normal glucose metabolism and promoting growth and development in chickens. Deficiency or imbalance in insulin production can lead to metabolic disorders and other health issues.

## Effects of BTG1 and BTG2 genes

**FIGURE 1:**
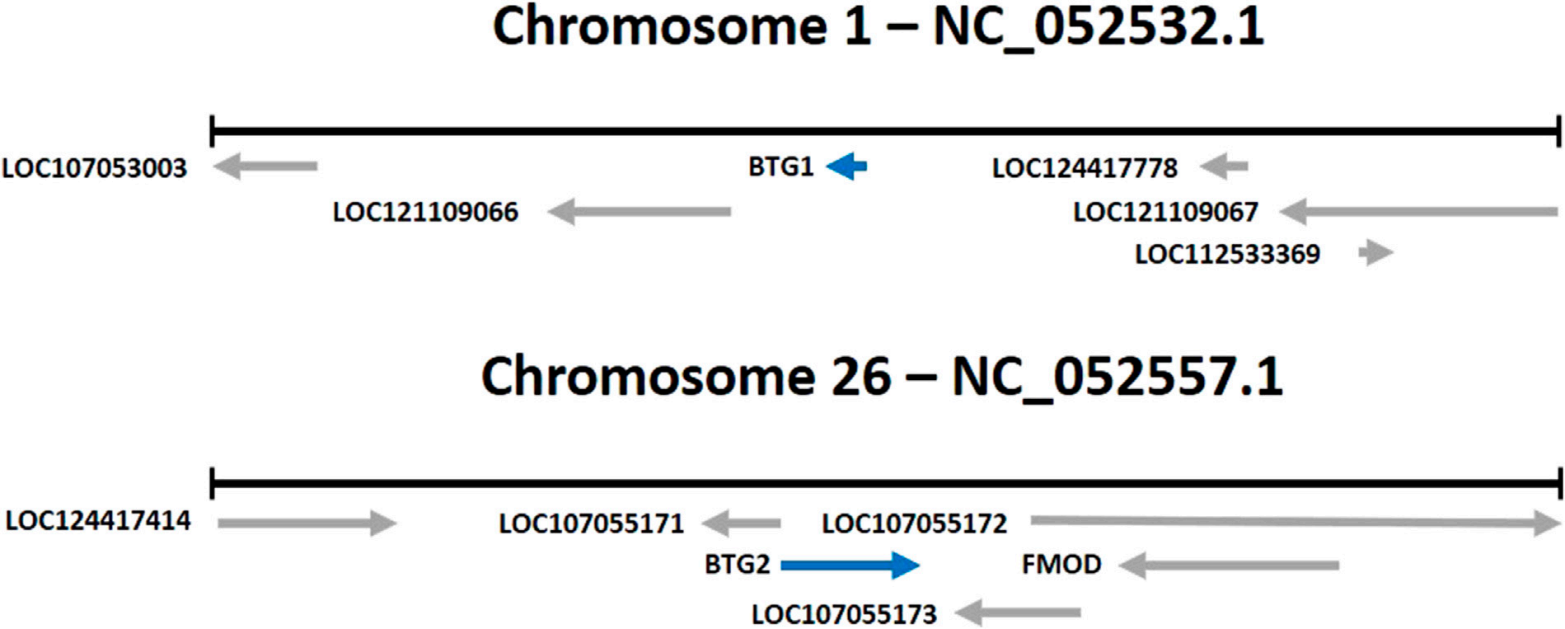
Location of the BTG1 and BTG2 genes (*Gallus gallus*).

As noted above, B-cell translocation gene 1 (BTG1) and BTG2 are two genes that belong to the BTG/Tob family of proteins (see Figure 1). These genes have been shown to play a role in regulating cell growth, differentiation, and apoptosis in various animal species, including chickens.

Studies have shown the expression of BTG1 and BTG2 genes in the brain and nervous system during embryonic development in chickens. This suggests that these genes may be involved in the regulation and function of neural development.

BTG1 has been shown to influence muscle growth in different animals (Rodier et al., 2001; Iwai et al., 2004; Busson et al., 2005; Yuniati et al., 2019). In a study of BTG2 gene expression levels in pigs, an association with inhibition of primary muscle fiber proliferation was found (Feng et al., 2007). The BTG1 gene was identified by genome-wide association studies (GWAS) as a promising marker for muscle development in Brahman cattle (Crispim et al., 2015). High expression of the BTG1 is found in immature muscle cells, especially during active muscle growth. It has been determined that BTG1 stimulates the activity of myogenic factors, in particular the nuclear receptors already known as positive regulators of myogenin, which boosts the transcription by myoblasts and stimulates their differentiation (Busson et al., 2005). In addition, BTG1 and BTG2 have been shown to participate in regulating muscle development and growth in chickens (Mohammadabadi et al., 2021). It was proven that BTG1 and BTG2 are abundantly expressed in skeletal muscles. There were also differences between broilers and laying hens in gene expression levels in the sternum, pancreas, and heart (Zhao et al., 2022). It can be assumed that when analyzing the nucleotide sequence of these genes, differences in polymorphic sites can be determined in meat- and egg-producing hens, which would indicate a direct relationship with productivity and the prospects of these genes as markers affecting metabolism.

Studies have observed that the increasing expression of these genes is related to upregulated muscle regeneration after injury, suggesting that they may be involved in the repair and growth of muscle tissue. Muscle stem cells (MuSCs) are a crucial part in the mechanism of muscle regeneration through paracrine signaling interactions. BTG2 promotes differentiation in hematopoietic and nerve cells by inhibiting Id3 and cyclin D1 to limit cell cycle progression (Yuniati et al., 2019). Scientists hypothesize that BTG2 and Id3 may be markers of resting-state transcription of MuSCs (de Micheli et al., 2020).

Furthermore, BTG1 and BTG2 have been implicated in the regulation of immune function in chickens. Some studies have detected that the high expression of these genes is manifested in response to viral infections, suggesting that they may participate in the host response to viral pathogens. The correlation of BTG2 with immune reactions was confirmed by a group of scientists, who proved that the expression of this gene is significantly correlated with tumor purity (Zhang et al., 2022). Some studies have identified an important role for the BTG1 and BTG2 genes in tumor suppression, i.e., a decreased expression of these genes results in more severe malignant neoplasms, leading to death. These genes may be biomarkers of disease progression (Cho et al., 2004; Kanda et al., 2015).

BTG1 and BTG2 play key roles in a wide range of cellular activities, including cell growth, proliferation, and apoptosis, through modulation of various central biological steps such as transcription, post-transcription, and translation (Kim et al., 2022). The researchers have found that activation of the expression of BTG1 and BTG2 by B-cells at the early stages of their development, especially in living organisms during the embryonic period and the period of postnatal adaptation, is associated with their regulatory functions, particularly their involvement in cell differentiation (Tijchon et al., 2016).

Overall, the BTG1 and BTG2 genes take part in regulating various biological processes in chickens, including neural development, muscle growth and regeneration, and immune function. It has been determined that BTG1 is required to preserve and replenish stem cells in the dentate gyrus and subventricular zone (SVZ) in adults. The reduced number of adult neurons in the dentate gyrus in BTG1-null mice is associated with subtle impairments in hippocampus-dependent learning and memory (Micheli et al., 2015).

Table 1 summarizes the functions of BTG1 and BTG2 that have been previously investigated.

**TABLE 1:** Functions of the BTG1 and BTG2 genes.

Functions of BTG1 and BTG2	Publications
Muscle growth and regeneration	Rodier et al. (2001), Iwai et al. (2004), Busson et al. (2005), Yuniati et al. (2019), Feng et al. (2007), Crispim et al. (2015), Mohammadabadi et al. (2021), and de Micheli et al. (2020)
Immune reactions	Zhang et al. (2022), Cho et al. (2004), and Kanda, et al. (2015)
Regulating glucose metabolism, insulin sensitivity, and resistance	Xiao et al. (2016), Lee et al. (2002), Kim et al. (2016), Kim et al. (2014), Takahashi et al. (2012), Hwang et al. (2013), McKinnon and Docherty (2001), and Zhao et al. (2022)
Cell growth, proliferation, apoptosis, and differentiation	Kim et al. (2022) and Tijchon et al. (2016)
Preserve and replenish stem cells	Micheli et al. (2015)

## Relationship between insulin and BTG1 and BTG2 genes

By analyzing the available literature, the possible pathways of the influence of BTG1 and BTG2 genes on insulin levels in chickens were identified.

In humans, insulin is the major anabolic hormone regulating the metabolism of glucose, fats, and proteins. The role of insulin is to maintain body weight either by decreasing catabolism or increasing anabolism (Heslin et al., 1996).

Some serious metabolic diseases are related to loss of insulin sensitivity by cells. This state has been called insulin resistance. Currently, the mechanisms of its development are not very clear and understandable. As stated above, BTG1, as an important cofactor, is involved in many physiological processes, so it may also be related with energy metabolism and exchange of glucose. *In vitro*, the expression of this gene can influence insulin signaling. So when mice with insulin resistance are infected by adenovirus, the high expression of BTG1 increases the sensitivity of cells to insulin, similar to that observed in wild-type mice. Moreover, transgenic mice with high levels of expression of BTG1 were insensitive to insulin resistance induced by a high-carbohydrate diet (Xiao et al., 2016).

Another study discovered that BTG2 expression was decreased in the adipose tissue of obese humans, proposing that it could be involved in the development of insulin resistance. In an experiment on mice, it was found that the expression level of BTG2 varies depending on the body build, and the knockdown of this gene increased lipid accumulation (Kim et al., 2016).

In addition, a new function of BTG1 in the regulation of insulin sensitivity in the liver cells in mice via activation of transcription factor JUN (c-Jun), which stimulates cell proliferation, has been observed. The study showed that the expression of BTG1 is increased in the rat liver with protein and energy deficiency in the diet (Lee et al., 2002).

Recent work has suggested that members of this family play a role in maintaining blood glucose homeostasis (Takahashi et al., 2012; Kim et al., 2014). High expression of BTG2 by mice accompanied the activation of the gluconeogenic gene in the liver, which leads to an increase in the level of glucose in the bloodstream and, later, a decrease in insulin tolerance (Xiao et al., 2016).

The regulation of insulin secretion also involves glucagon-like peptide-1 (GLP-1), which is synthesized by the endocrine cells of the intestines. This peptide hormone belongs to the incretin family and is an important element in the treatment of type-2 diabetes. Recent research studies established a novel molecular mechanism that proves that GLP-1 stimulates the expression of the insulin gene via BTG2 (Hwang et al., 2013).

The critical stage of pancreatic development is characterized by the high expression levels of pancreatic duodenal homeobox-1 (PDX1), and in the case of the low expression level of PDX1, the pancreas could not progress well. So homozygous mutation of this gene in mice and humans leads to agenesis of the pancreas. In adults, PDX1 manifested by the beta cells of the islets of Langerhans is related to the synthesis of insulin, and that expressed by delta cells activates the secretion of somatostatin, which ultimately leads to decreased glucose levels. Expression of PDX1 is also observed in the dispersed endocrine cells of the duodenum. The reason for some types of diabetes, for example, MODY4 in young people and type II (non-insulin-dependent) diabetes, which usually manifests itself at an older age, may be heterozygous mutations in the PDX1 gene, which can worsen glucose tolerance (McKinnon et al., 2001).

Scientists have found that BTG2 may regulate the expression of PDX1 in pancreatic β-cells, so its stimulation may lead to the secretion of insulin. As noted above, infection by adenovirus leads to a high level of expression of BTG2, which is activated significantly, increasing insulin secretion and expression of the PDX1 gene. More detailed molecular studies showed that BTG2 had a direct influence on PDX1, increasing PDX1 occupancy at the insulin gene promoter. Increased insulin secretion was stimulated by GLP1 or its analog, Exendin-4 (Ex-4), which is used in the treatment of diabetes and was blocked by the small interfering RNA system of BTG2.

The level of expression of BTG1 and BTG2 genes in chickens depends on the breeds and type of the tissue. So the expression of BTG genes in thoracic and pancreatic cells was negatively correlated with the glucose level in the bloodstream of chickens (Zhao et al., 2022).

Further research is needed to fully understand the mechanisms of the effects of BTG1 and BTG2 genes on insulin and glucose metabolism.

## Discussion

Based on the analytic results of many sources, we can confidently assume that the use of BTG1 and BTG2 as candidate genes for regulation of the insulin level is promising and can lead to significant results in the poultry sector, where insulin is one of the main hormones responsible for regulation of metabolism, increase in amino acid transport, and stimulation of lipogenesis.

All studies conducted have addressed the effects of the expression of these genes, but they have not analyzed the amino acid sequence or searched for associations with the level of genetic expression.

The study of promoters and coding regions of these genes and the search for their single-nucleotide polymorphisms may provide a more accurate understanding of the mechanisms of their functioning.

For this work, we need to sequence these genes and collect expression data from chickens under equal rearing conditions. Once the associations have been made, we will be able to determine the relationship of the nucleotide sequence with the level of synthesis of the proteins under study.

This work will open the possibility of using these genes as candidates for MAS of chickens.

These research studies can provide a significant boost to the work regarding avian feed conversion, and the obtained data may improve our understanding of the functioning of the relationship between BTG1 and BTG2 genes, insulin levels, and metabolic regulation.

The study of this topic is a promising area for physiologists and geneticists who are researching the functions of the pancreas and insulin.
